# Further rationale for optimal combined modality treatments

**DOI:** 10.18632/oncotarget.16117

**Published:** 2017-03-10

**Authors:** Robert J. Griffin, Ruud P.M. Dings, Issam Makhoul

**Affiliations:** Department of Radiation Oncology Radiation Biology Division, University of Arkansas for Medical Sciences, Little Rock, AR, USA

**Keywords:** radiotherapy, anti-angiogenic agent, sunitinib, re-oxygenation

The recent report in Oncotarget by Kleibeuker et al. “Low dose angiostatic treatment counteracts radiotherapy induced tumor perfusion and enhances the anti-tumor effect” is a useful new perspective for our improved understanding of how angiostatic treatment regimens may best be incorporated into conventional ‘standard of care’ practices. Indeed, the results presented in this recent report support and re-emphasize results that we and others have reported [1, 2]. The addition of certain angiogenic inhibitors during and after radiotherapy could improve tumor control, however until now many of these studies used only single radiation doses. In general, more clinically relevant fractionated regimens were found to be impacted to a greater degree than single dose regimens- a finding that is borne out and expanded upon in the new data presented by Kleibeuker et al.

In particular, the concept that treatment with an anti-angiogenic agent (and its timing) has marked effects on the physiology of solid tumors, which in turn dictates the response to chemotherapy or radiation therapy, is important to examine. It has long been hypothesized that due to the relatively long time periods across which many standard chemotherapy and radiation therapy regimens are implemented (i.e., 6-8 weeks), the effects of anti-angiogenic treatment could either be synergistic or antagonistic to the patient outcomes [3]. Markers of response to an anti-angiogenic treatment can be detected throughout a treatment regimen, as in a recent clinical study at our center that employed Avastin against breast tumors [4, 5]. However, we do not necessarily know if the anti-angiogenic treatments are improving or possibly antagonizing response to chemoradiation along the way [6]. For example, if the tumor blood flow decreases significantly after treatment with an anti-angiogenic agent, subsequent radiation treatment or chemotherapy access may be rendered ineffective or much less useful than anticipated since tumor hypoxia may have become much more extensive by the time that the next dose of conventional therapy is administered. Certainly, the opposite may be true if blood flow is increased before drug or radiation exposure, or if the anti-angiogenic agent is applied after the conventional treatment regimen has ended. The traditional thinking in radiotherapy has been that the ‘re-oxygenation’ that may occur over the course of a fractionated treatment regimen will lead to improved overall control of the tumor by radiation due to enhanced sensitivity created by the improved oxygenation profile. Whether or not this truly occurs in the majority of human tumors is still a matter of debate, yet the current work suggests a measure of re-oxygenation and reperfusion after radiation exposure. However, the distinctive finding here is that using sunitinib to block the reperfusion is effective in tumor control, clearly better than radiation alone in the limited fraction regimen that was studied.

The authors of the current report go on to demonstrate that a very powerful logic may exist when combining these approaches. Namely, the rebound effect that occurs during and after fractionated radiotherapy, the classical understanding of solid tumor re-oxygenation after radiotherapy, can be positively impacted (i.e., inhibited) by treating with low dose antiangiogenic agent at the appropriate time during the therapy regimen. Generally this would be during the last part of the fractionation scheme and likely to continue for a period of time after radiation concludes to ensure that there is a limited or non-existent rebound of perfusion and viability in the tumor. However, the authors do not elaborate about the differential effect of radiotherapy on different components of the tumor (cancer cells, vasculature, fibroblasts and immune cells), all of which may play a role in the indirect effects of radiotherapy on the tumor. For example, some of the radiotherapy effect may be related to endothelial cell injury. The initial ischemia that follows and the direct effect of radiotherapy on tumor cells leads to upregulation of proangiogenic factors, improved perfusion and repopulation. Mechanistically, it is intriguing to speculate that the suppressed rebound effect was mediated by blocking the recruitment of circulating endothelial progenitor cells [7]. In addition, the recognized capability of sunitinib to enhance the anti-tumor immune response in recent literature could be playing a role in Kleibeuker's results and those of other groups and should not be ignored as the approach is further developed [8]. A key feature of the approach may be in the relatively low or sub-optimal dose (although still significant when scaled to humans) of sunitinib that was applied, with a rationale to block the reperfusion effect but not cause excessive vascular damage or hypoxia.

Although the field of anti-angiogenic agents can be seen as ‘old news’ to many, in reality there is still much to be gained in the appropriate scheduling of combination therapy- something that is frequently ignored to an extent in design and reporting of clinical studies. These new results reinforce the importance of applying multi-modality treatments in a fashion that exploits the best that each treatment has to offer. Certainly, the idea of controlling or inhibiting an angiogenic reaction to radiation-induced changes in physiology has its place as a realistic strategy to consider for better management of a variety of solid tumors.
